# Dr. Amos Westcott

**Published:** 1873-10

**Authors:** 


### OBITUARY.

Dr. Amos Westcott.—At a meeting of the Dentists of Syracuse, N. Y. relative to the loss the dental profession has sustained in the sudden demise of Dr. Westcott, the following preamble and resolutions were unanimously adopted; [Dr. Jas. Chandler, Chairman, and Dr. F. D. Nellis, Secretary of the meeting :]

" *Whereas*, a mysterious Providence has removed from our midst Amos Westcott, M. D., D. D. S., one of the oldest dentists of this city; therefore,

*Resolved*, That in the death of Dr. Westcott, the profession of dentistry has lost one of its foremost members,—one who has done as much as any member of the profession to promote its advancement and growth,—who, both as a writer and teacher, instilled into the minds of students the most valuable principles of our practice, the beneficial effects of which are as extensive as civilization itself, and whose name is honored by dentists throughout the world.

*Resolved*, That while we testify to the pre-eminence enjoyed by the deceased in the dental profession, we bear witness with equal emphasis to his worth and uprightness as a man. Genial in manner, he always stood ready to assist, by his counsel, those who were struggling upwards, taking a deep interest in the welfare of his fellow-men, and especially of the city of his adoption.

*Resolved*, That we deeply sympathize with the afflicted family in their sad bereavement.

*Resolved*, That, as a further mark of respect, we, the dentists of Syracuse, attend the funeral of the deceased in a body.

*Resolved*, That a copy of these resolutions be transmitted to the family of the deceased, and that the same be published in the daily papers of this city and in the leading dental journals of the country."

Dr. Westcott was for a number of years connected with the Faculty of the Baltimore College of Dental Surgery, being Professor of Theory and Practice of Dental Surgery from 1846 to 1850, previous to which he was Demonstrator of Operative Dentistry, having received the diploma of this institution in 1843.

Dr. Westcott, after his return to Syracuse, was elected to the responsible position of mayor of the city, and was regarded as one of the leading dental practitioners in the State of New York.

